# Supplementary material for: Control of bacterial cell wall autolysins by peptidoglycan crosslinking mode
Source: Nat Commun. 2024 Sep 11;15:7937. doi: 10.1038/s41467-024-52325-2 (PMC11390936; doi:10.1038/s41467-024-52325-2)
Supplement: Supplementary file 1 — Supplementary Information [file 41467_2024_52325_MOESM1_ESM.pdf]

SUPPLEMENTARY INFORMATION

Control of bacterial cell wall autolysins by peptidoglycan crosslinking mode

Laura Alvarez[1#], Sara B. Hernandez[1,a#], Gabriel Torrens[1], Anna I. Weaver[2,3,b], Tobias Dörr[2,3,4] and Felipe Cava[1,5,6,7,*].

Affiliations:

[1]Department of Molecular Biology, Umeå University, Umeå, Sweden.

[2]Department of Microbiology, Cornell University, Ithaca, New York, USA.

[3]Weill Institute for Cell and Molecular Biology, Cornell University, Ithaca, New York, USA.

[4]Cornell Institute of Host-Microbe Interactions and Disease, Cornell University, Ithaca, New York, USA.

[5]Umeå Center for Microbial Research (UCMR), Umeå University, Umeå, Sweden.

[6]The Laboratory for Molecular Infection Medicine Sweden (MIMS), Umeå, Sweden.

[7]Science for Life Laboratory (SciLifeLab), Umeå University, Umeå, Sweden.

Current affiliation:

[a]Instituto de Bioquímica Vegetal y Fotosíntesis, Consejo Superior de Investigaciones Científicas and Universidad de Sevilla, Seville, Spain.

[b]Department of Microbiology, Blavatnik Institute, Harvard Medical School, Boston, MA, USA.

[#]These authors contributed equally.

***For correspondence:** felipe.cava@umu.se

Lead contact: Felipe Cava

Running title: Autolysin inhibition by peptidoglycan crosslinking

Keywords: cell wall, *Vibrio cholerae*, peptidoglycan, LD-crosslink, anhydromuropeptides, lytic transglycosylases

Table of contents:

- Supplementary Figures 1-12
- Supplementary Table 1: Bacterial strains
- Supplementary Table 2: Plasmids
- Supplementary Table 3: Oligonucleotides
- Supplementary Table 4. List of phage genomes and endolysins.
- Supplementary References

Supplementary Figures

a

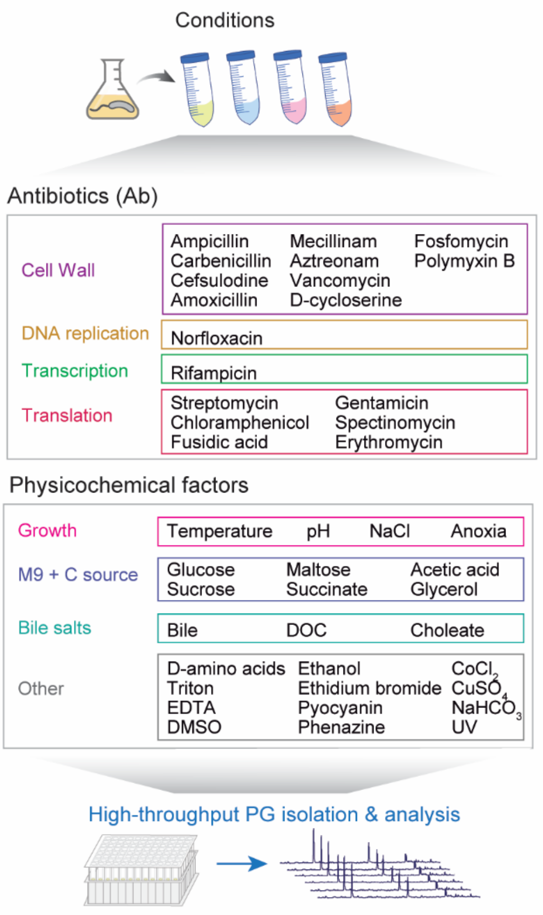

b

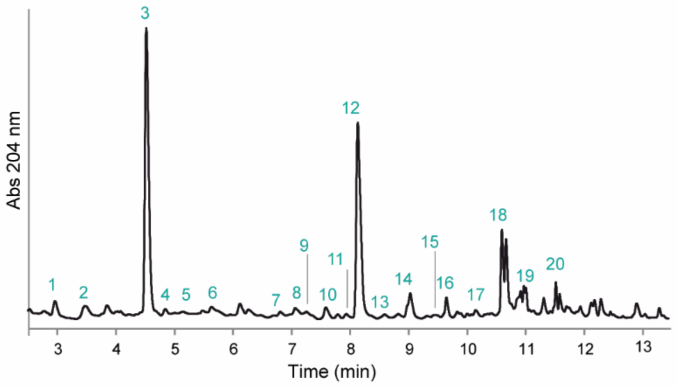

c

Peak	Name	Structure
1	M3	GlcNAc-MurNAc-L-Ala-D-Glu-DAP
2	$M4^{G}$	GlcNAc-MurNAc-L-Ala-D-Glu-DAP-Gly
3	M4	GlcNAc-MurNAc-L-Ala-D-Glu-DAP-D-Ala
4	M2	GlcNAc-MurNAc-L-Ala-D-Glu
5	M5	GlcNAc-MurNAc-L-Ala-D-Glu-DAP-D-Ala-D-Ala
6	M3-Lpp	GlcNAc-MurNAc-L-Ala-D-Glu-DAP-Braun's lipoprotein (TK residues)
7	$M3^{Anh}$	GlcNAc-(1,6-anhydro)MurNAc-L-Ala-D-Glu-DAP
8	$D44^{G}$	M4-$M4^{G}$ (crosslinked at D-Ala-DAP)
9	D43	M4-M3 (crosslinked at D-Ala-DAP)
10	D34	M3-M4 (crosslinked at DAP-DAP)
11	$M4^{M}$	GlcNAc-MurNAc-L-Ala-D-Glu-DAP-D-Met
12	D44	M4-M4 (crosslinked at D-Ala-DAP)
13	D45	M4-M5 (crosslinked at D-Ala-DAP)
14	$M4^{Anh}$	GlcNAc-(1,6-anhydro)MurNAc-L-Ala-D-Glu-DAP-D-Ala
15	T344	M3-M4-M4 (crosslinked at DAP-DAP and at D-Ala-DAP)
16	T444	M4-M4-M4 (crosslinked at D-Ala-DAP)
17	$D34^{Anh}$	M3-$M4^{Anh}$ (crosslinked at DAP-DAP)
18	$D44^{Anh}$	M4-$M4^{Anh}$ (crosslinked at D-Ala-DAP)
19	$T444^{Anh}$	M4-M4-$M4^{Anh}$ (crosslinked at D-Ala-DAP)
20	$D44^{2xAnh}$	$M4^{Anh}$-$M4^{Anh}$ (crosslinked at D-Ala-DAP)

Supplementary Figure 1. Screening of *V. cholerae*'s peptidoglycan plasticity across different conditions. a. Wild type *V. cholerae* was exposed to sub-MIC concentrations of the indicated antibiotics or stress conditions (panels on the left). Several substances were tested at different concentrations such that a total of 110 different growth conditions were surveyed. Samples were processed for PG isolation and analysis using a filter-based high-throughput preparation method. **b.** Representative chromatogram of wild type *V. cholerae* strain. Identified peaks are listed. **c.** Table of muropeptides identified in **b**, confirmed by mass spectrometry. GlcNAc: *N*-acetyl glucosamine; MurNAc: *N*-acetyl muramic acid; (1,6-anhydro)MurNAc: terminal 1,6-anhydro-*N*-acetyl muramic acid; L-Ala: L-alanine; D-Glu: D-glutamic acid; DAP: *meso* diaminopimelic acid; D-Ala: D-alanine; D-Met: D-methionine; Gly: glycine; Lpp: Braun's lipoprotein.

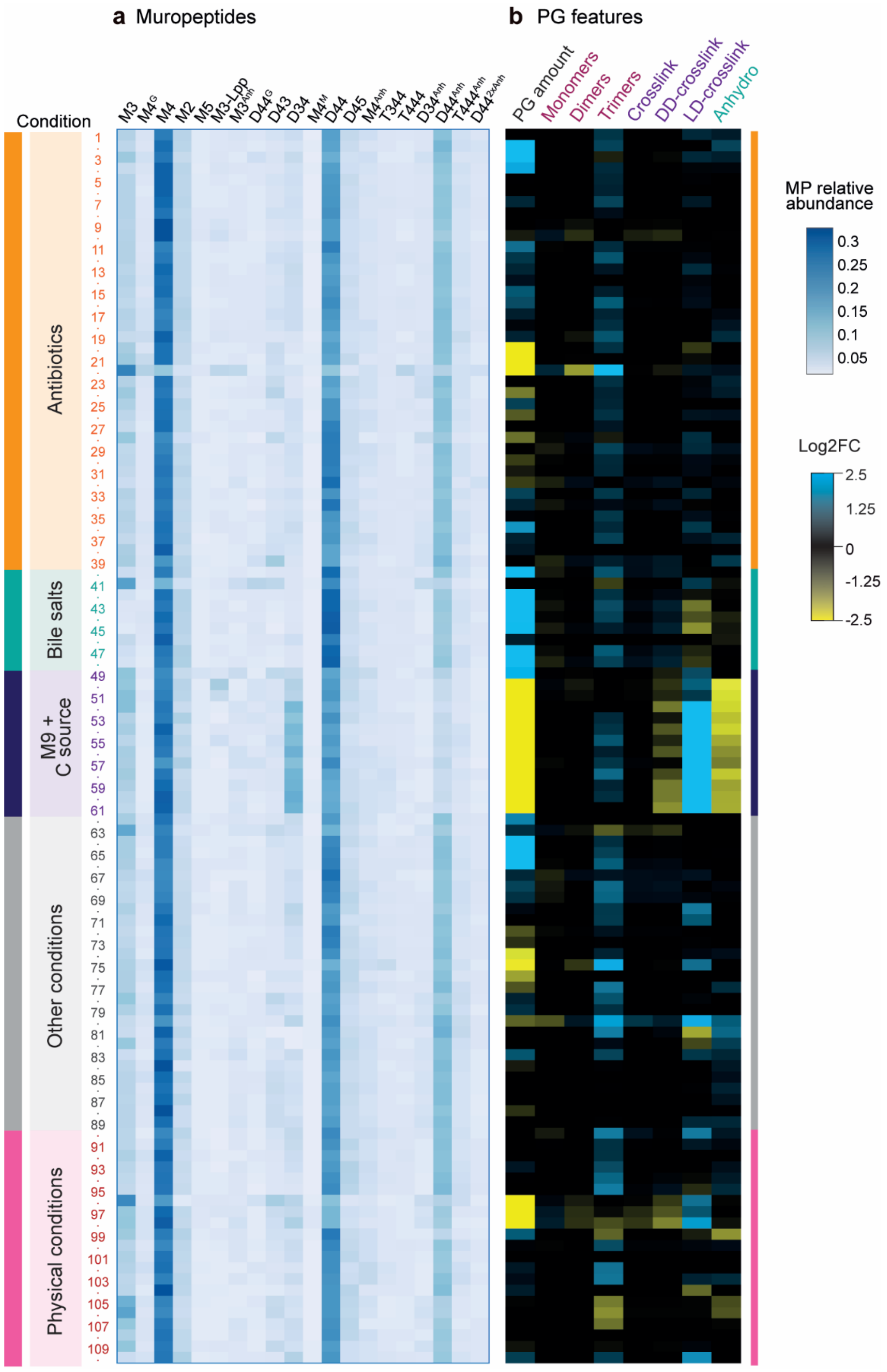

Supplementary Figure 2. Peptidoglycan analysis of *V. cholerae* grown under different conditions. a. Heatmap representing the relative abundance of the identified muropeptides

(MP) for all tested conditions (Suppl. Data 1). **b.** Heatmap representing the Log2FC calculated for the main peptidoglycan (PG) features. All values are mean of 3 biological replicates. M9+C source: M9 minimal medium supplemented with carbon sources. Details and source data are provided in Supplementary Data 1.

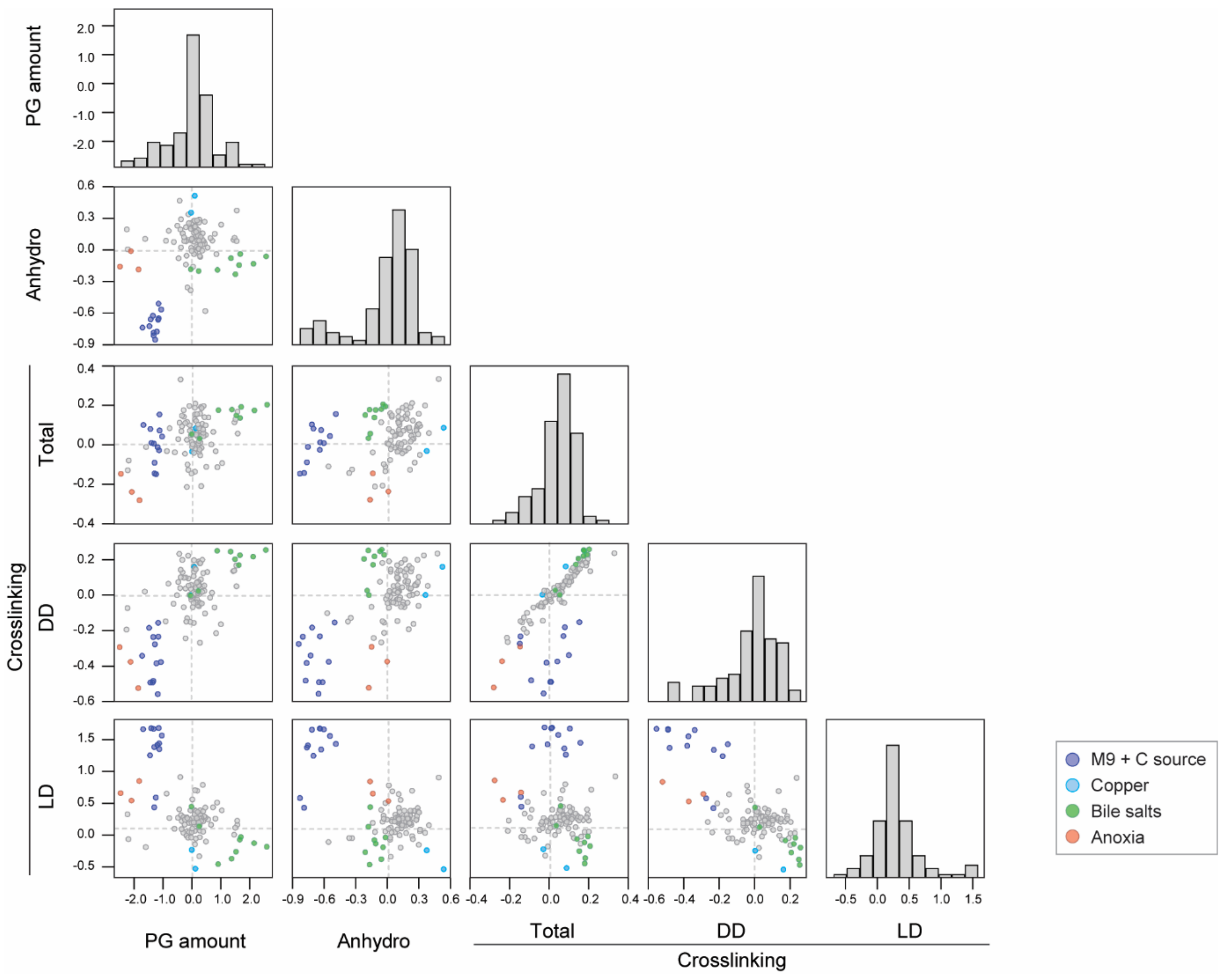

Supplementary Figure 3. Scatter matrix for the main peptidoglycan features for *V. cholerae* grown under the tested conditions. Scatter plots representing the Log2FC of main peptidoglycan (PG) features: relative PG amount, anhydromuropeptide levels, crosslinking (total and type). Clusters of conditions of interest are highlighted. Values shown in scatter plots are the mean of 3 biological replicates. Histograms showing the distribution of samples for every dimension are represented in the diagonal. Source data are provided in Supplementary Data 1.

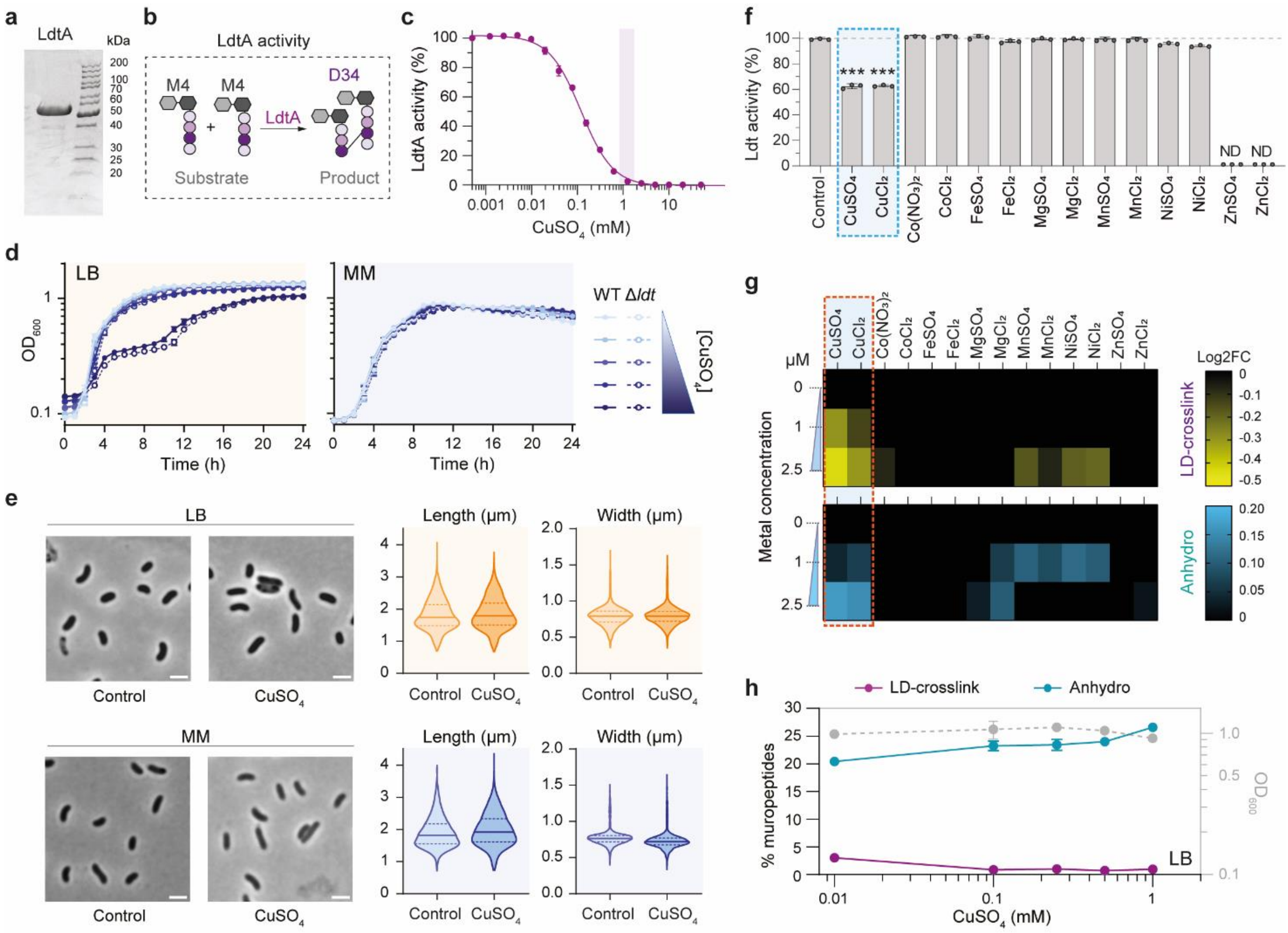

Supplementary Figure 4. Inhibition of *V. cholerae* LD-transpeptidase by copper. a. Purified LdtA from *V. cholerae*, the LD-transpeptidase which produces LD-crosslinks. The molecular weight of the purified His-tagged protein is predicted to be 58.8 kDa. **b.** Schematic diagram of the LdtA in vitro reaction: LdtA converts M4 monomers into LD-crosslinked D34 dimers. **c.** Relative LdtA activity at different copper concentrations. Working concentration in bacterial cultures in LB is highlighted (1 mM). In vitro assays were performed in triplicate. Data are presented as mean values +/- standard deviation. **d.** Growth of *V. cholerae* WT and Δ*ldt* mutant in LB with 0.5-2 mM $CuSO_4$ and MM with 1-10 µM $CuSO_4$. Growth curves were performed in triplicate. Data are presented as mean values +/- standard deviation. **e.** Representative phase contrast images and violin plots of the length and mean width of *V. cholerae* WT grown in LB (top) or MM (bottom) in the presence or absence of $CuSO_4$ at 1

mM (in LB) or 5 µM (in MM). Scale bar: 2 µm. Samples size: LB control, n = 6493; LB $CuSO_4$, n = 4144; MM control, n = 4724; MM $CuSO_4$, n = 3821. **f.** Relative LdtA activity in presence of 0.1 mM of different metal salts. In vitro assays were performed in triplicate. Data are presented as mean values +/- standard deviation. Statistical significance was determined using unpaired t-tests, with an alpha level of 0.05. Two-tailed p values are reported in the Source Data file. ND, not detected; ***, p <0.001. **g.** Effect of different metal salts on *V. cholerae* PG features in vivo. Heatmaps represent the mean Log2FC calculated for LD-crosslink and anhydromuropeptide levels in the PG of *V. cholerae* cultures grown in MM with 1 or 2.5 µM of respective metal salt. PG analyses were performed in triplicate. **h.** Concentration-dependent effect of copper on LD-crosslink and anhydromuropeptide levels in the PG of *V. cholerae* grown in LB. Assays were performed in quintuplicate. Data are presented as mean values +/- standard deviation. Source data are provided as a Source Data file.

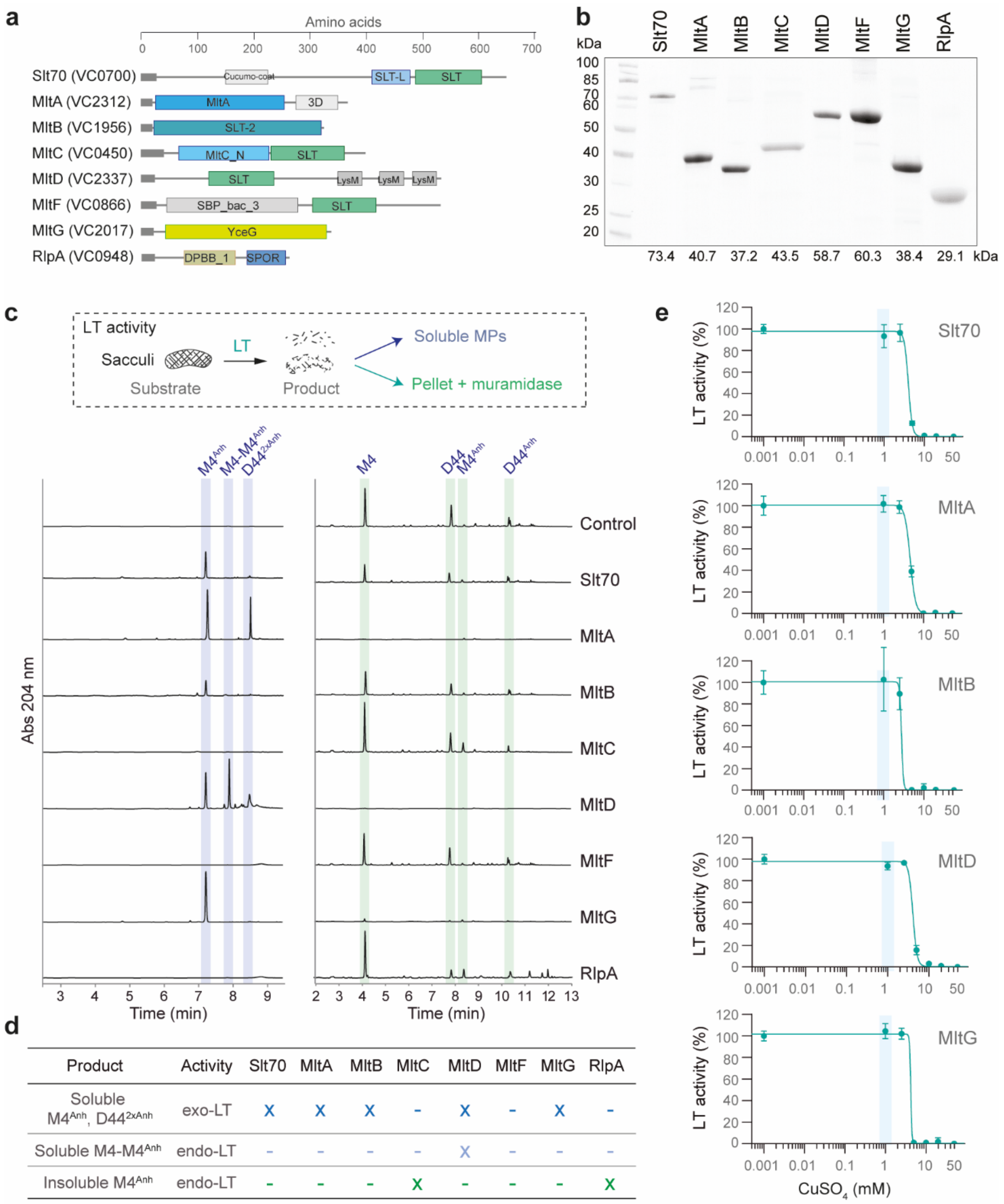

Supplementary Figure 5. In vitro lytic transglycosylase activity. a. Domain architecture of the lytic transglycosylases (LTs) in *V. cholerae*. **b.** Purified LTs from *V. cholerae*. Predicted molecular weight of the purified His-tagged proteins is indicated at the bottom. **c.** Schematic diagram of in vitro assay used to study the activity of *V. cholerae*'s LTs. Sacculi from *V. cholerae* WT grown in LB was used as substrate. Upon digestion with the LTs, the soluble

products were directly analyzed by LC, and the insoluble pellets were further digested with muramidase prior to LC analysis. Major products are highlighted in the chromatograms. **d.** Summary table of the LT activities of each tested enzyme. **e.** Relative LT activity at different copper concentrations of the LTs. Working concentration in bacterial cultures in LB is highlighted (1 mM). In vitro assays were performed in triplicate. Data are presented as mean values +/- standard deviation. Source data are provided as a Source Data file.

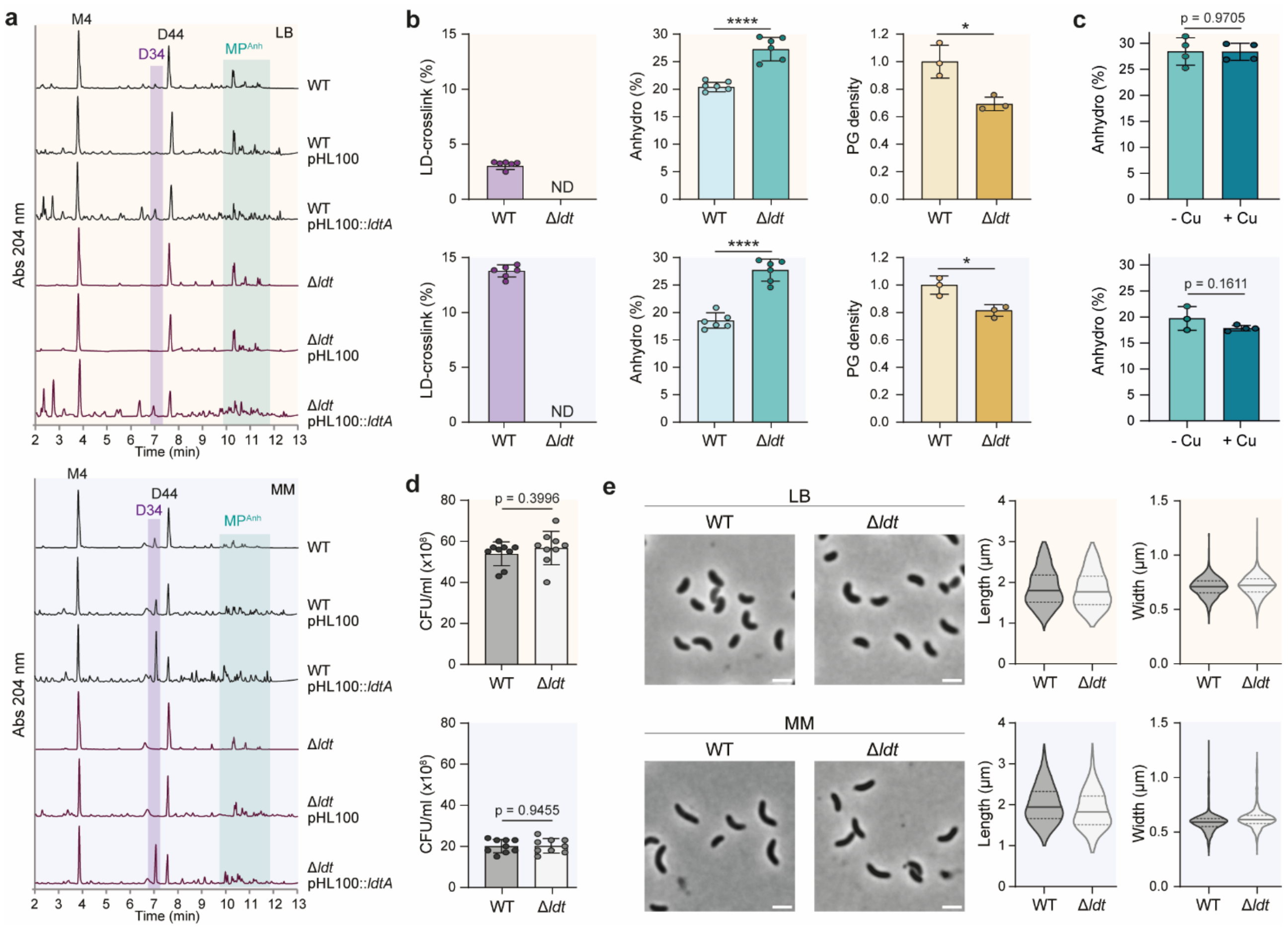

Supplementary Figure 6. Characterization of the *V. cholerae* Δ*ldt* mutant. a. Representative chromatograms obtained from PG of *V. cholerae* WT, Δ*ldt* mutant and complemented strains grown in LB (top) or MM (bottom). LD-crosslinked D34 muropeptide and anhydromuropeptides (MP^{Anh}) are indicated. **b.** Relative amount of LD-crosslinked muropeptides, anhydromuropeptides and PG density of *V. cholerae* WT and Δ*ldt* strains grown in LB (top) or MM (bottom). PG analyses were performed in 6 replicates (triplicates for PG density). Data are presented as mean values +/- standard deviation. Statistical significance was determined using unpaired t-tests, with an alpha level of 0.05. Two-tailed p values are reported in the Source Data file. ND, not detected; *, $p < 0.05$; ****, $p < 0.0001$. **c.** Relative amount of anhydromuropeptides in the PG of the *V. cholerae* Δ*ldt* mutant in the presence of $CuSO_4$ 1 mM $CuSO_4$ in LB (top) or 5 µM $CuSO_4$ in MM (bottom). PG analyses

were performed in quadruplicates. Data are presented as mean values +/- standard deviation. Statistical significance was determined using unpaired t-tests, with an alpha level of 0.05. Two-tailed p values are reported. **d.** CFU/ml of *V. cholerae* WT and Δ*ldt* mutant cultures grown overnight in LB (top) or MM (bottom). 9 replicates were analyzed in the viability assays. Data are presented as mean values +/- standard deviation. Statistical significance was determined using unpaired t-tests, with an alpha level of 0.05. Two-tailed p values are reported. **e.** Representative phase contrast images and violin plots of the length and mean width of *V. cholerae* WT and Δ*ldt* mutant cells grown in LB (top) or MM (bottom). Scale bar: 2 µm. Samples size: WT LB, n = 1807; Δ*ldt* LB, n = 1630; WT MM, n = 1418; Δ*ldt* MM, n = 1264. Source data are provided as a Source Data file.

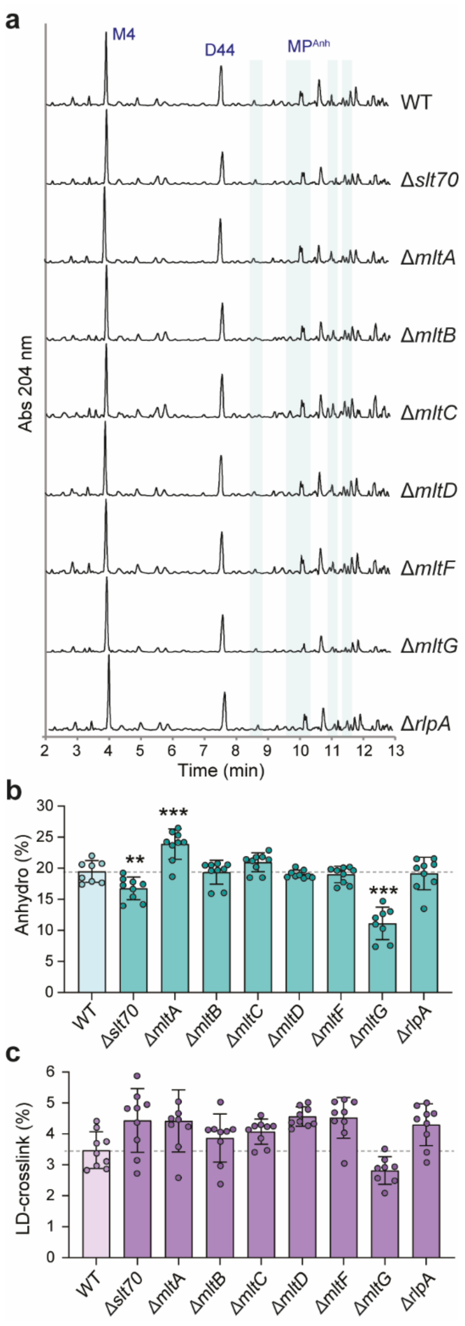

Supplementary Figure 7. Peptidoglycan analysis of *V. cholerae* lytic transglycosylase mutants. a. Representative chromatograms of the PG of *V. cholerae* WT and ΔLT strains grown in LB. Anhydromuropeptides (MP^{Anh}) are indicated in green. **b.** Relative amount of anhydromuropeptides in the PG of *V. cholerae* WT and LT mutants grown in LB. **c.** Relative amount of LD-crosslink in the PG of *V. cholerae* WT and ΔLT strains grown in LB. PG analyses were performed in 9 replicates. Data are presented as mean values +/- standard deviation. Statistical significance was determined using unpaired t-tests, with an alpha level

of 0.05. Two-tailed p values are reported in the Source Data file. **, $p < 0.01$; ***, $p < 0.001$.

Source data are provided as a Source Data file.

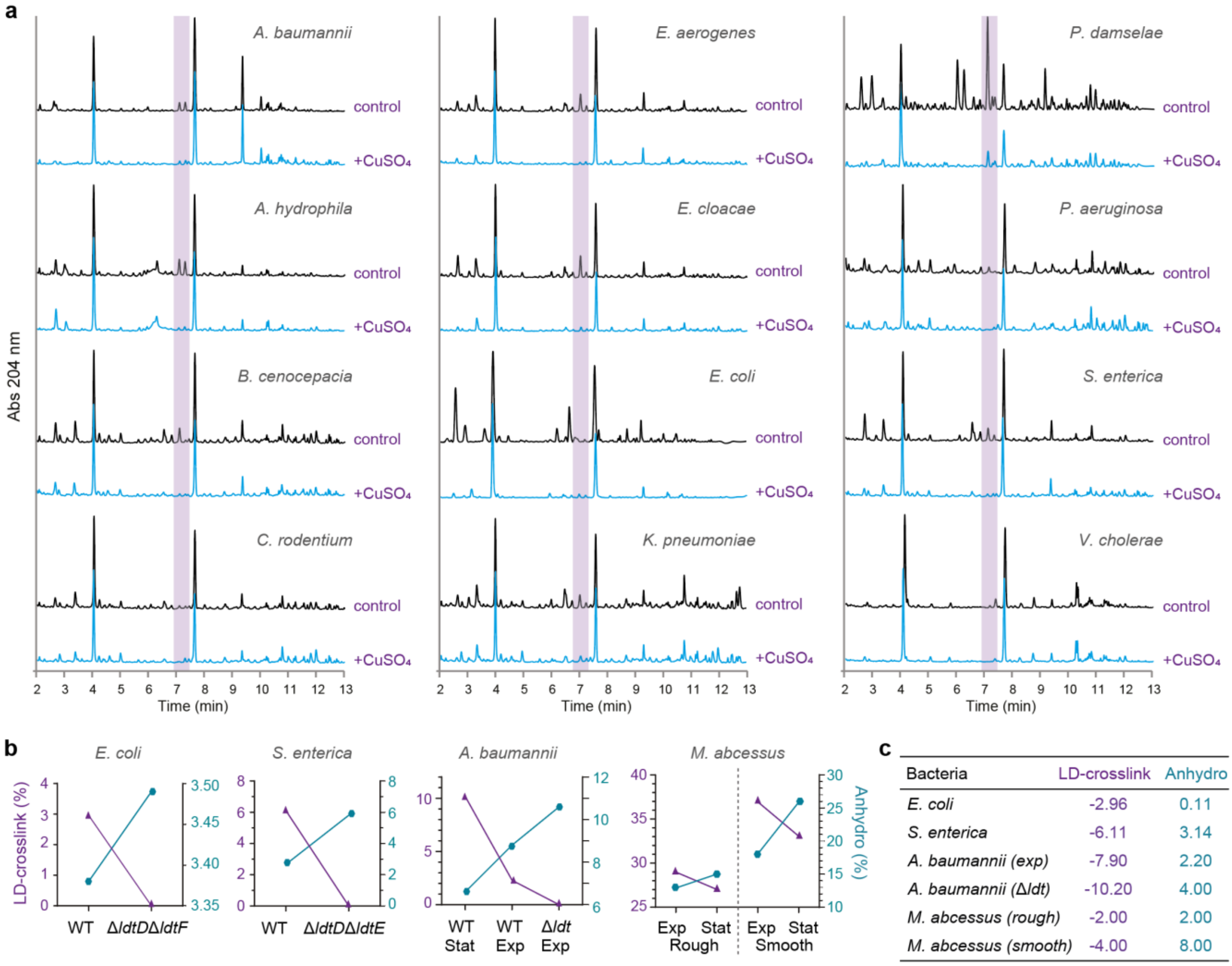

Bacteria	LD-crosslink	Anhydro
E. coli	-2.96	0.11
S. enterica	-6.11	3.14
A. baumannii (exp)	-7.90	2.20
A. baumannii (Δldt)	-10.20	4.00
M. abcessus (rough)	-2.00	2.00
M. abcessus (smooth)	-4.00	8.00

Supplementary Figure 8. Effect of inhibition of LD-transpeptidases by copper in different bacterial species. a. Representative chromatograms of different bacterial species (*Acinetobacter baumannii, Aeromonas hydrophila, Burkholderia cenocepacia, Citrobacter rodentium, Enterobacter aerogenes, Enterobacter cloacae, Escherichia coli, Klebsiella pneumoniae, Photobacterium damselae, Pseudomonas aeruginosa, Salmonella enterica* and *Vibrio cholerae*) grown in absence (control) or presence of 1 mM $CuSO_4$. All bacteria were grown in LB, except for *P. damselae* which was grown in TSB. **b.** Inverse correlation between relative amounts of LD-crosslink and anhydromuropeptides observed for different bacteria in the literature [1-4]. Exp, exponential phase; Stat, stationary phase. **c.** Calculated

change in the relative amounts of LD-crosslink and anhydromuropeptides from the data in **b**. Source data are provided as a Source Data file.

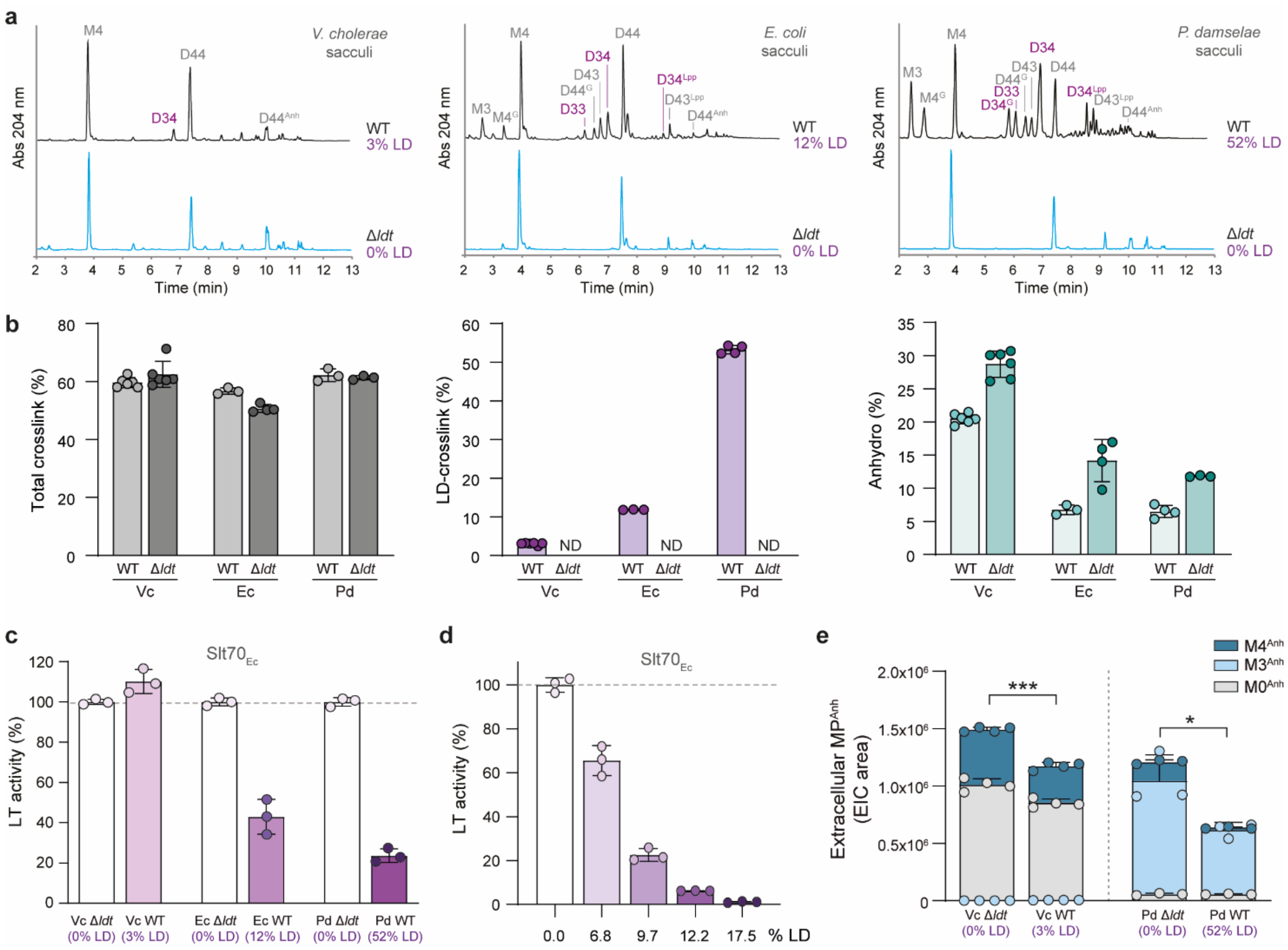

Supplementary Figure 9. LD-crosslinks inhibit the activity of endogenous lytic transglycosylases. a. Representative chromatograms of *V. cholerae, E. coli* and *P. damselae* WT and Δ*ldt* mutant sacculi used in the in vitro reactions to test LT activity. Percentage of LD-crosslinking is indicated. **b.** Relative total crosslink, LD-crosslinking and anhydromuropeptide levels in the WT and Δ*ldt* mutant sacculi used as substrate in the in vitro reactions. PG analyses were performed in 6 replicates for *V. cholerae*, 3-4 replicates for *E. coli*, and 3-4 replicates for *P. damselae*. Data are presented as mean values +/- standard deviation. **c.** Relative LT activity of Slt70 from *E. coli* ($Slt70_{Ec}$) on *V. cholerae* (Vc), *E. coli* (Ec) or *P. damselae* (Pd) WT or Δ*ldt* mutant sacculi. Activity is calculated relative to the Δ*ldt* sacculi substrate, with 0% LD-crosslinks. In vitro assays were performed in triplicates. Data are presented as mean values +/- standard deviation. **d.** Relative LT activity

of Slt70 from *E. coli* on substrate with indicated LD-crosslinking levels. Activity is calculated relative to the sacculi substrate with 0% LD-crosslinks. In vitro assays were performed in triplicates. Data are presented as mean values +/- standard deviation. **e.** Detection of extracellular anhydromuropeptides (product of exo-LT activity) in the growth medium of the *V. cholerae* and *P. damselae* WT and ∆*ldt* strains. Analyses were performed in quadruplicates for *V. cholerae* and triplicates for *P. damselae*. Data are presented as mean values +/- standard deviation. Statistical significance was determined using unpaired t-tests, with an alpha level of 0.05. Two-tailed p values are reported in the Source Data file. *, $p < 0.05$; ***, $p < 0.001$. Source data are provided as a Source Data file.

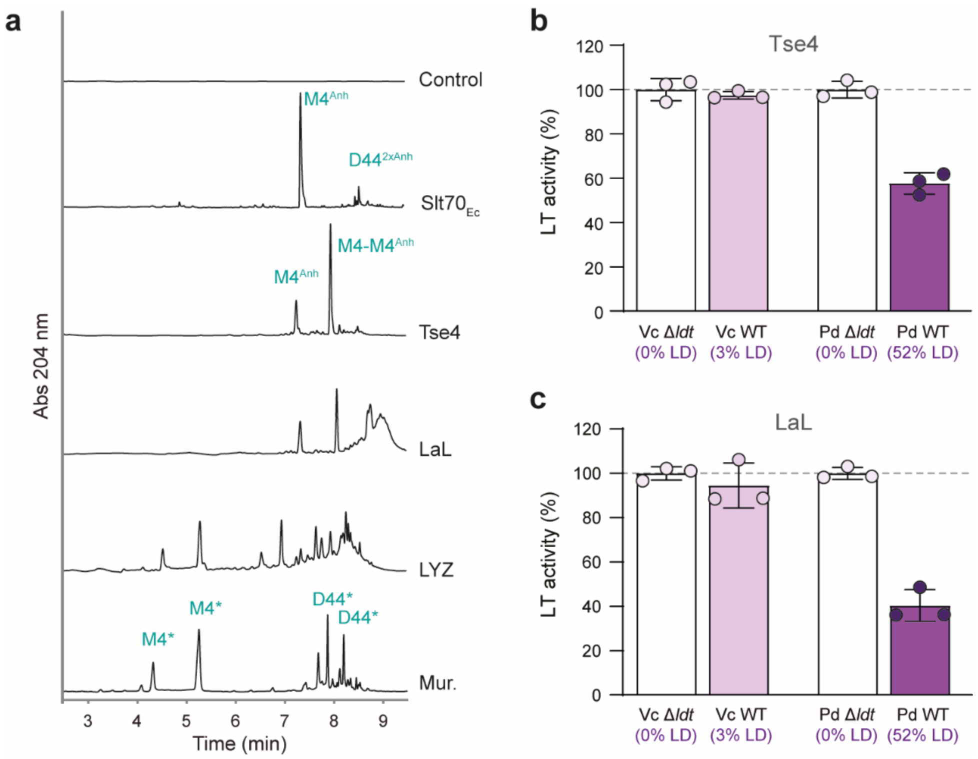

Supplementary Figure 10. LD-crosslinks inhibit the activity of predatory lytic transglycosylases. a. Representative chromatograms showing the released muropeptides after digestion of *V. cholerae* sacculi with Slt70 from *E. coli* ($Slt70_{Ec}$), Tse4 from *A. baumannii,* bacteriophage lambda endolysin (LaL), chicken egg white lysozyme (LYZ) and mutanolysin from *Streptomyces globisporus* (Mur.). **b.** Relative LT activity of Tse4 on *V. cholerae* or *P. damselae* WT or Δ*ldt* mutant sacculi. Activity is calculated relative to the Δ*ldt* sacculi substrate, with 0% LD-crosslinks. **c.** Relative LT activity of LaL on *V. cholerae* or *P. damselae* WT or Δ*ldt* mutant sacculi. Activity is calculated relative to the Δ*ldt* sacculi substrate, with 0% LD-crosslinks. In vitro assays were performed in triplicates. Data are presented as mean values +/- standard deviation. Source data are provided as a Source Data file.

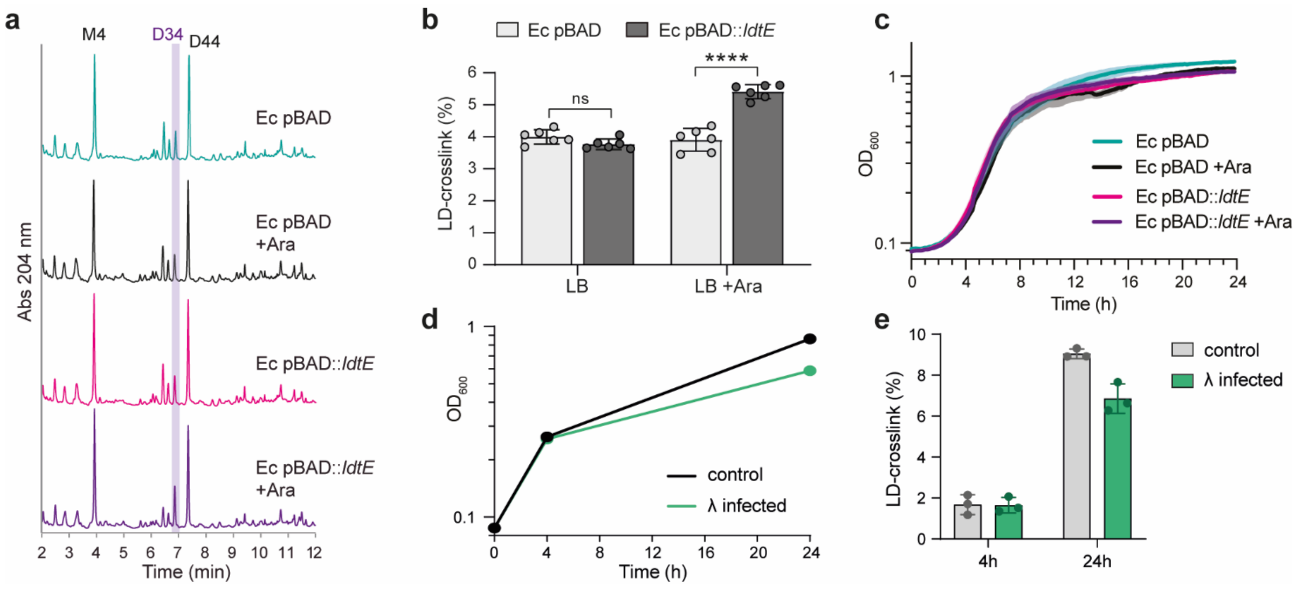

Supplementary Figure 11. LD-crosslink levels in the peptidoglycan of *E. coli* subjected to phage infections. a. Representative chromatograms obtained from the PG of *E. coli* (Ec) JM109 with empty pBAD or pBAD::*ldtE* (for overexpression of the LD-transpeptidase LdtE) grown with and without inducer (arabinose 0.2%, +Ara). **b.** Quantification of LD-crosslinking in the PG of *E. coli* JM109 with empty pBAD or pBAD::*ldtE*, grown with and without inducer. PG analyses were performed in 6 replicates. Data are presented as mean values +/- standard deviation. Statistical significance was determined using unpaired t-tests, with an alpha level of 0.05. Two-tailed p values are reported in the Source Data file. ns: not significant; ****, $p < 0.0001$. **c.** Growth curves of *E. coli* JM109 with empty pBAD or pBAD::*ldtE*, grown with and without inducer. Assays were performed in triplicate. Data are presented as mean values +/- standard deviation. **d.** Optical density (OD_{600}) of *E. coli* JM109 infected with lambda phage and time points for PG sample collection. Assays were performed in triplicate. Data are presented as mean values +/- standard deviation. **e.** Variation in LD-crosslinking in *E. coli* JM109 infected or not with lambda phage. Assays were performed in triplicate. Data are presented as mean values +/-

standard deviation. Paired t-test results indicate differences are not significant (two-tailed p value = 0.4914). Source data are provided as a Source Data file.

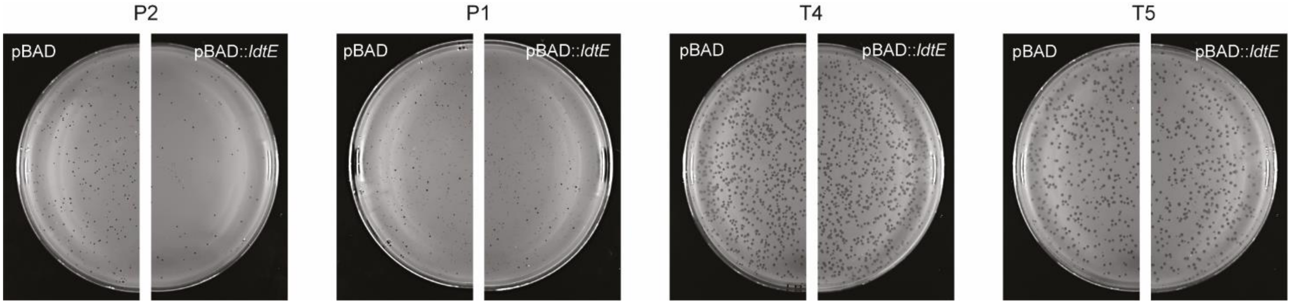

Supplementary Figure 12. Increased LD-crosslinking specifically provides resistance to LT-encoding phages. Representative plates showing phage plaque formation upon infection of *E. coli* JM109 carrying empty pBAD or pBAD::*ldtE* with P2 (encoding an LT-like endolysin), P1 and T4 (encoding lysozyme-like endolysins), or T5 (encoding an endopeptidase) phages. LB agar plates are supplemented with 20 µg/ml chloramphenicol, 10 mM $MgSO_4$, and 0.2% (w/v) arabinose.

Supplementary Tables.

Supplementary Table 1. Bacterial strains.

**Bacteria**	**Media**	**Temp (º C)**	**Source/Ref**
Vibrio cholerae C6706	LB	37	[5]
Vibrio cholerae N16961	LB	37	[6]
Vibrio cholerae N16961 Δ*ldt* (Δ*ldtA* Δ*ldtB*)	LB	37	[7]
Vibrio cholerae N16961 Δ*slt70*	LB	37	[8]
Vibrio cholerae N16961 Δ*mltA*	LB	37	[8]
Vibrio cholerae N16961 Δ*mltB*	LB	37	[8]
Vibrio cholerae N16961 Δ*mltC*	LB	37	[9]
Vibrio cholerae N16961 Δ*mltD*	LB	37	[8]
Vibrio cholerae N16961 Δ*mltF*	LB	37	[8]
Vibrio cholerae N16961 Δ*mltG*	LB	37	[9]
Vibrio cholerae N16961 Δ*rlpA*	LB	37	[9]
Escherichia coli DH5α	LB	37	*E. coli* genetic stock
Escherichia coli BL21	LB	37	*E. coli* genetic stock
Escherichia coli BW25113	LB	37	[10]
Escherichia coli BW25113 Δ*ldt (*Δ*ldtA* Δ*ldtB* Δ*ldtC* Δ*ldtD* Δ*ldtE* Δ*ldtF*)	LB	37	[11]
Escherichia coli C600	LB	37	*E. coli* genetic stock
Escherichia coli JM109	LB	37	*E. coli* genetic stock
Acinetobacter baumannii ATCC-17978	LB	37	ATCC collection
Aeromonas hydrophila ATCC-7966	LB	37	ATCC collection
Burkholderia cenocepacia K56-2	LB	37	Courtesy of M. Valvano
Citrobacter rodentium ATCC-51116	LB	37	ATCC collection
Enterobacter aerogenes DSM-30053	LB	30	DSMZ collection
Enterobacter cloacae DSM-30054	LB	30	DSMZ collection
Klebsiella pneumoniae MKP103	LB	37	Courtesy of C. Manoil
Photobacterium damselae CIP102761	TSB	30	Courtesy of B. Gomez-Gil
Photobacterium damselae CIP102761 Δ*ldt (*Δ*ldtA1* Δ*ldtA2* Δ*ldtB*)	TSB	30	Alvarez et al, unpublished
Pseudomonas aeruginosa PA14	LB	37	Courtesy of G. Pier
Salmonella enterica serovar Typhimurium ATCC-14028	LB	37	ATCC collection

Supplementary Table 2. Plasmids.

Plasmid	Description or use	Source/Ref
pHL100	Plasmid for protein expression in *V. cholerae* under the control of the P_{lac} promoter. KnR.	[7]
pHL100::*ldtA*	pHL100 derivative. Expression of LdtA. KnR.	[7]
pET15b	Expression vector with T7 promoter and terminator flanking MCS, and optional N-terminal 6xHis-tag sequence. AmpR.	Novagen
pET22b	Expression vector with T7 promoter and terminator flanking MCS, pelB leader sequence for potential periplasmic localization and optional C-terminal 6xHis-tag sequence. AmpR.	Novagen
pET28b::*ldtA*	pET28b derivative. Production and purification of LdtA-His protein. KnR.	[7]
pET28b::*slt70Ec*	pET28b derivative. Production and purification of *E. coli*'s Slt70-His protein. KnR.	[12]
pET15b::*His-mltA*	pET15b derivative. Production and purification of His-MltA. AmpR.	This work
pET15b::*His-mltB*	pET15b derivative. Production and purification of His-MltB. AmpR.	This work
pET15b::*His-mltC*	pET15b derivative. Production and purification of His-MltC. AmpR.	This work
pET15b::*His-mltD*	pET15b derivative. Production and purification of His-MltD. AmpR.	This work
pET15b::*His-mltF*	pET15b derivative. Production and purification of His-MltF. AmpR.	This work
pET15b::*His-mltG*	pET15b derivative. Production and purification of His-MltG. AmpR.	This work
pET15b::*His-rlpA*	pET15b derivative. Production and purification of His-RlpA. AmpR.	This work
pET22b::*slt70-His*	pET22b derivative. Production and purification of Slt70-His. AmpR.	This work
pET22b::*LaL-His*	Production and purification of LaL-His (lambda lysozyme). AmpR.	This work
pBAD33	Expression vector with P_{BAD} promoter. CmR.	[13]
pBAD::*ldtE*	pBAD33 derivative. Overexpression of *E. coli*'s LDT LdtE. CmR.	Akbar Espaillat, unpublished.

Supplementary Table 3. Oligonucleotides.

FCP_ID	Name	Sequence	Description
FCP5421	mltA_NdeI_fw	**aaaaCATATG**CAACCTAACGATCGTGCTCAG	Cloning MltA into pET15b, Δ19 aa in N-terminus, NdeI restriction site
FCP5422	mltA_BamHI_rv1	**aaaaGGATCC**CTATTGCTGTTTTTCCGGCGG	Cloning MltA into pET15b, contains Stop codon, BamHI restriction site
FCP5424	mltB_NdeI_fw	**aaaaCATATG**AATGAAGTCAGTTTTGAACAATATGTCG	Cloning MltB into pET15b, Δ19 aa in N-terminus, NdeI restriction site
FCP5425	mltB_BamHI_rv1	**aaaaGGATCC**TTAGAACGCAATCCGATCCG	Cloning MltB into pET15b, contains Stop codon, BamHI restriction site
FCP5427	mltC_NdeI_fw	**aaaaCATATG**GAATTTATCGAGAAAATTTACGATGTTGATT	Cloning MltC into pET15b, Δ39 aa in N-terminus, NdeI restriction site
FCP5428	mltC_BamHI_rv1	**aaaaGGATCC**TTATCCCGCGTTAAATTCCTTCTTAAAT	Cloning MltC into pET15b, contains Stop codon, BamHI restriction site
FCP5430	mltD_NdeI_fw	**aaaaCATATG**ACCACATCAGATGACCAAGCG	Cloning MltD into pET15b, Δ24 aa in N-terminus, NdeI restriction site
FCP5431	mltD_BamHI_rv1	**aaaaGGATCC**TTATGCGCTGAATTTGGTCACATC	Cloning MltD into pET15b, contains Stop codon, BamHI restriction site
FCP5433	mltF_XhoI_fw1	**aaaaCTCGAG**GATTCCGAGCCCAAAAGCG	Cloning MltF into pET15b, Δ27 aa in N-terminus, XhoI restriction site
FCP5434	mltF_BamHI_rv1	**aaaaGGATCC**CTAATTTTTGCTCTCAGTGGATGG	Cloning MltF into pET15b, contains Stop codon, BamHI restriction site
FCP5437	mltG_NdeI_fw	**aaaaCATATG**TATGTTGTTAAGCAGATGGATCAGTA	Cloning MltG into pET15b, Δ21 aa in N-terminus, NdeI restriction site

FCP_ID	Name	Sequence	Description
FCP5438	mltG_XhoI_rv1	**aaaaCTCGAG**TCATTGTTTTG TTCTAAGTTTTTTGAGATAA	Cloning MltG into pET15b, contains Stop codon, XhoI restriction site
FCP5440	rlpA_NdeI_fw	**aaaaCATATG**TATGATATGTC TGACGATCAAGCAC	Cloning RlpA into pET15b, Δ23 aa in N-terminus, NdeI restriction site
FCP5441	rlpA_BamHI_rv1	**aaaaGGATCC**TCATTTAGCA CGTTTATTAATCGTCTT	Cloning RlpA into pET15b, contains Stop codon, BamHI restriction site
FCP5710	slt_NdeI_fw2	**aaaaCATATG**ACGCGCCTGA CGGTATTTAAGC	Cloning Slt70 into pET22b, from Start codon, NdeI restriction site
FCP5420	slt_BamHI_rv2	**aaaaGGATCC**GAATACTTTG TATTTAACTCATGCTCATT	Cloning Slt70 into pET22b, no Stop codon, BamHI restriction site
FCP5849	LAL_NdeI_fw	**aaaaCATATG**GTAGAAATCA ATAATCAACGTAAGGC	Cloning LaL into pET22b, from Start codon, NdeI restriction site
FCP5850	LAL_BamHI_rv	**aaaaGGATCC**GATACATCAA TCTCTCTGACCGTTCC	Cloning LaL into pET22b, no Stop codon, BamHI restriction site

Supplementary Table 4. List of phage genomes and endolysins.

Phage	ICTV family	Genome[a]	Endolysin[b]	UniProt, CDD domain
Lambda	*Siphoviridae*	NC_001416	NP_040645.1	lambda_lys-like
P2	*Myoviridae*	NC_001895.1	NP_046765.1	lambda_lys-like
P1	*Myoviridae*	NC_005856.1	YP_006484.1	lyz_P1
T4	*Myoviridae*	NC_000866.4	NP_049736.1	T4-like_lys
T5	*Demerecviridae*	NC_005859.1	YP_006868.1	L-Ala-D-Glu_peptidase_like

[a] GenBank nucleotide accession number
[b] GenBank protein accession number

Supplementary References

1 Hernandez, S. B., Cava, F., Pucciarelli, M. G., Garcia-Del Portillo, F., de Pedro, M. A. & Casadesus, J. Bile-induced peptidoglycan remodelling in Salmonella enterica. *Environmental microbiology* **17**, 1081-1089 (2015). https://doi.org:10.1111/1462-2920.12491

2 Kang, K. N. *et al.* Septal Class A Penicillin-Binding Protein Activity and ld-Transpeptidases Mediate Selection of Colistin-Resistant Lipooligosaccharide-Deficient Acinetobacter baumannii. *mBio* **12** (2021). https://doi.org:10.1128/mBio.02185-20

3 Lavollay, M. *et al.* The peptidoglycan of Mycobacterium abscessus is predominantly cross-linked by L,D-transpeptidases. *J Bacteriol* **193**, 778-782 (2011). https://doi.org:10.1128/JB.00606-10

4 More, N. *et al.* Peptidoglycan Remodeling Enables Escherichia coli To Survive Severe Outer Membrane Assembly Defect. *mBio* **10** (2019). https://doi.org:10.1128/mBio.02729-18

5 Thelin, K. H. & Taylor, R. K. Toxin-coregulated pilus, but not mannose-sensitive hemagglutinin, is required for colonization by Vibrio cholerae O1 El Tor biotype and O139 strains. *Infect Immun* **64**, 2853-2856 (1996). https://doi.org:10.1128/iai.64.7.2853-2856.1996

6 Heidelberg, J. F. *et al.* DNA sequence of both chromosomes of the cholera pathogen Vibrio cholerae. *Nature* **406**, 477-483 (2000). https://doi.org:10.1038/35020000

7 Cava, F., de Pedro, M. A., Lam, H., Davis, B. M. & Waldor, M. K. Distinct pathways for modification of the bacterial cell wall by non-canonical D-amino acids. *EMBO J* **30**, 3442-3453 (2011). https://doi.org:10.1038/emboj.2011.246

8 Weaver, A. I. *et al.* Lytic transglycosylases mitigate periplasmic crowding by degrading soluble cell wall turnover products. *eLife* **11** (2022). https://doi.org:10.7554/eLife.73178

9 Weaver, A. I., Jimenez-Ruiz, V., Tallavajhala, S. R., Ransegnola, B. P., Wong, K. Q. & Dorr, T. Lytic transglycosylases RlpA and MltC assist in Vibrio cholerae daughter cell separation. *Mol Microbiol* **112**, 1100-1115 (2019). https://doi.org:10.1111/mmi.14349

10 Datsenko, K. A. & Wanner, B. L. One-step inactivation of chromosomal genes in Escherichia coli K-12 using PCR products. *Proceedings of the National Academy of Sciences of the United States of America* **97**, 6640-6645 (2000). https://doi.org:10.1073/pnas.120163297

11 Kuru, E. *et al.* Fluorescent D-amino-acids reveal bi-cellular cell wall modifications important for Bdellovibrio bacteriovorus predation. *Nature microbiology* **2**, 1648-1657 (2017). https://doi.org:10.1038/s41564-017-0029-y

12 Espaillat, A. *et al.* Chemometric Analysis of Bacterial Peptidoglycan Reveals Atypical Modifications That Empower the Cell Wall against Predatory Enzymes and Fly Innate Immunity. *J Am Chem Soc* **138**, 9193-9204 (2016). https://doi.org:10.1021/jacs.6b04430

13 Guzman, L. M., Belin, D., Carson, M. J. & Beckwith, J. Tight regulation, modulation, and high-level expression by vectors containing the arabinose PBAD promoter. *J Bacteriol* **177**, 4121-4130 (1995). https://doi.org:10.1128/jb.177.14.4121-4130.1995
