# Supplementary material for: Control of bacterial cell wall autolysins by peptidoglycan crosslinking mode
Source: Nat Commun. 2024 Sep 11;15:7937. doi: 10.1038/s41467-024-52325-2 (PMC11390936; doi:10.1038/s41467-024-52325-2)
Supplement: Supplementary file 3 — Description of Additional Supplementary Files [file 41467_2024_52325_MOESM3_ESM.pdf]

<u>Description of Additional Supplementary Files</u>

File Name: Supplementary Data 1.
Description: PG analysis of the bacterial pathogen *Vibrio cholerae* across different conditions. The file includes the list of conditions used in the screening; a description of the analyzed PG features; the relative amount, fold change and log2 fold change (Log2FC) values for the PG features and identified muropeptides.
