# Supplementary material for: Control of bacterial cell wall autolysins by peptidoglycan crosslinking mode
Source: Nat Commun. 2024 Sep 11;15:7937. doi: 10.1038/s41467-024-52325-2 (PMC11390936; doi:10.1038/s41467-024-52325-2)
Supplement: Supplementary file 5 — Reporting Summary [file 41467_2024_52325_MOESM5_ESM.pdf]

nature portfolio

Corresponding author(s): Felipe Cava

Last updated by author(s): Aug 23, 2024

Reporting Summary

Nature Portfolio wishes to improve the reproducibility of the work that we publish. This form provides structure for consistency and transparency in reporting. For further information on Nature Portfolio policies, see our Editorial Policies and the Editorial Policy Checklist.

Statistics

For all statistical analyses, confirm that the following items are present in the figure legend, table legend, main text, or Methods section.

n/a	Confirmed	
☐	☒	The exact sample size (*n*) for each experimental group/condition, given as a discrete number and unit of measurement
☐	☒	A statement on whether measurements were taken from distinct samples or whether the same sample was measured repeatedly
☐	☒	The statistical test(s) used AND whether they are one- or two-sided<br>*Only common tests should be described solely by name; describe more complex techniques in the Methods section.*
☒	☐	A description of all covariates tested
☐	☒	A description of any assumptions or corrections, such as tests of normality and adjustment for multiple comparisons
☐	☒	A full description of the statistical parameters including central tendency (e.g. means) or other basic estimates (e.g. regression coefficient) AND variation (e.g. standard deviation) or associated estimates of uncertainty (e.g. confidence intervals)
☐	☒	For null hypothesis testing, the test statistic (e.g. *F*, *t*, *r*) with confidence intervals, effect sizes, degrees of freedom and *P* value noted<br>*Give P values as exact values whenever suitable.*
☒	☐	For Bayesian analysis, information on the choice of priors and Markov chain Monte Carlo settings
☒	☐	For hierarchical and complex designs, identification of the appropriate level for tests and full reporting of outcomes
☒	☐	Estimates of effect sizes (e.g. Cohen's *d*, Pearson's *r*), indicating how they were calculated

Our web collection on statistics for biologists contains articles on many of the points above.

Software and code

Policy information about availability of computer code

Data collection	No custom code was generated for this study. Biotek Gen5 [v.08] was used to collect OD measurements. Waters Empower 3.6 and Waters UNIFI 1.8.1 were used for LC and LC-MS and MS/MS data acquisition, respectivelly. Zeiss Zen Blue edition [v2.0.0.0] software was used for collection of microscopy data.
Data analysis	No custom code was generated for this study. Graphpad Prism 8.0 was used for graphing and analyzing most data. Microscopy images were analyzed with Fiji/ImageJ [v1.53] and MicrobeJ plugin. Collected UPLC chromatographic data were analyzed using the PG-metrics pipeline in MATLAB R2023b (PMID: 29040278). Heatmaps were generated in R v 4.3 using the ggplot2 and pheatmap (https://github.com/raivokolde/pheatmap) packages. Waters UNIFI 1.8.1 was used for LC-MS and MS/MS data analysis.

For manuscripts utilizing custom algorithms or software that are central to the research but not yet described in published literature, software must be made available to editors and reviewers. We strongly encourage code deposition in a community repository (e.g. GitHub). See the Nature Portfolio guidelines for submitting code & software for further information.

Data

Policy information about availability of data

All manuscripts must include a data availability statement. This statement should provide the following information, where applicable:

- Accession codes, unique identifiers, or web links for publicly available datasets
- A description of any restrictions on data availability
- For clinical datasets or third party data, please ensure that the statement adheres to our policy

All data supporting the findings of this study are presented either in the paper, Supplementary Information or Source data file. The PG profile dataset from the conditions screening of V. cholerae generated in this study is provided in the Supplementary Data 1 file and has been deposited in the Figshare repository, with DOI: 10.6084/m9.figshare.26807920. Source data are provided with this paper. All strains are available upon request.

Research involving human participants, their data, or biological material

Policy information about studies with human participants or human data. See also policy information about sex, gender (identity/presentation), and sexual orientation and race, ethnicity and racism.

Reporting on sex and gender	N/A
Reporting on race, ethnicity, or other socially relevant groupings	N/A
Population characteristics	N/A
Recruitment	N/A
Ethics oversight	N/A

Note that full information on the approval of the study protocol must also be provided in the manuscript.

Field-specific reporting

Please select the one below that is the best fit for your research. If you are not sure, read the appropriate sections before making your selection.

☒ Life sciences ☐ Behavioural & social sciences ☐ Ecological, evolutionary & environmental sciences

For a reference copy of the document with all sections, see nature.com/documents/nr-reporting-summary-flat.pdf

Life sciences study design

All studies must disclose on these points even when the disclosure is negative.

Sample size	The sample size was not predetermined or calculated by statistical methods. We have followed common standard practice in the molecular biology and microbiology fields, choosing the sample size based on literature (e.g., PMID: 31289173) and variability observed in previous experience in the laboratory. We have typically used at least three biological replicates to account for random variation. This provided sufficient statistical power to rule out differences due to inherent biological variation. Only for measurement of bacterial width and length we have chosen larger sample sizes.
Data exclusions	No data were excluded from the study and analyses.
Replication	Experiments described in the manuscript were fully replicated, with three or more biological replicates. Where chromatograms or microscopy images are shown, these are representative of three biological replicates.
Randomization	No specific randomization processes were necessary as the experimental outcome does not depend on the order in which samples were analyzed in the experiments. Appropriate controls were used in all assays. In all experiments, control and experimental groups were done in isogenic strains.
Blinding	Blinding was not performed as all measurements in the study are quantitative at defined timepoints, and knowing the order or identity of a sample does not affect the results.

Reporting for specific materials, systems and methods

We require information from authors about some types of materials, experimental systems and methods used in many studies. Here, indicate whether each material, system or method listed is relevant to your study. If you are not sure if a list item applies to your research, read the appropriate section before selecting a response.

Materials & experimental systems

n/a	Involved in the study
☒	☐ Antibodies
☒	☐ Eukaryotic cell lines
☒	☐ Palaeontology and archaeology
☒	☐ Animals and other organisms
☒	☐ Clinical data
☒	☐ Dual use research of concern
☒	☐ Plants

Methods

n/a	Involved in the study
☒	☐ ChIP-seq
☒	☐ Flow cytometry
☒	☐ MRI-based neuroimaging

Plants

Seed stocks	N/A
Novel plant genotypes	N/A
Authentication	N/A
